# Adult-onset mast cell activation syndrome following scombroid poisoning: a case report and review of the literature

**DOI:** 10.1186/s13256-021-03190-w

**Published:** 2021-12-18

**Authors:** Isabelle Brock, Nicole Eng, Anne Maitland

**Affiliations:** 1 Comprehensive Asthma and Allergy, 200 S Broadway Suite 104, Tarrytown, NY 10591 USA; 2 Qolify, 99 Wall St Suite 1147, New York, NY 10005 USA; 3 Department of Neurology, Mount Sinai- South Nassau Hospital, The Chiari-EDS Center, 1420 Broadway, Hewlett, NY 11557 USA; 4 Department of Medical and Molecular Genetics, Indiana University School of Medicine, 975 W. Walnut Street, IB 130, Indianapolis, IN 46202 USA; 5 Nicole Eng, BS, Biomedical Studies, University of Pittsburgh, Pittsburgh, USA

**Keywords:** Mast cell, Mast cell activation syndrome, Scombroid poisoning, Histamine, Tryptase, Epithelium, Tolerance, Immune homeostasis

## Abstract

**Background:**

Mast cells are closely associated with epithelium, serving as sentinels responsible for the recognition of tissue injury and coordination of the initial inflammatory response. Upon detection of the injured cell content, mast cells then tailor the release of preformed and newly produced chemical mediators to the detected challenge, via an array of pathogen receptors. In addition to immunoglobulin E receptor-triggered mast cell activation, commonly referred to as allergic or atopic disorders, non-immunoglobulin E receptor mediated mast cell activation follows engagement of toll-like receptors, immunoglobulin G receptors, and complement receptors. Upon containment of the extrinsic challenge, acute inflammation is downregulated, and repair of the injured tissue ensues. The mast cell compartments must return to a baseline steady state to remain tolerant towards self-antigens and harmless entities, including environmental conditions, to prevent unnecessary immune activation and chronic hypersensitivity disorders. Over the past 50 years, an increasing number of patients are experiencing episodes of aberrant mast cell activation, not associated with allergen-specific mast cell disease or systemic mastocytosis. This led to proposed diagnostic criteria of mast cell activation syndrome. Mast cell activation syndrome is a heterogeneous disorder, defined by a combination of (1) recurrent symptoms typical of mast cell activation, (2) an increase of validated mast cell derived mediators, and (3) response to treatment with mast cell stabilizing or mast cell mediator-targeted therapies. Onset of mast cell activation syndrome ostensibly reflects the loss of tolerance in the mast cell compartment to nonthreatening entities and nonhazardous environmental conditions. The etiology of chronic mast cell dysregulation and associated intolerance to self-antigens or harmless entities is not well understood, but a growing number of studies point to exposure of the epithelial borders, which leads to inappropriate or excessive mast cell activation or impaired resolution of acute inflammation following neutralization of the identified pathogen.

**Case presentation:**

Here we present a case of adult onset mast cell activation syndrome following scombroid poisoning. Scombroid toxicity is usually a self-limited illness, but there are individuals who have been shown to have severe symptoms or persistent illness following histamine fish poisoning. We describe a 74-year-old Caucasian woman, with a history of drug-induced urticaria, who developed a constellation of hypersensitivity illnesses consistent with the diagnosis of mast cell activation syndrome after ingestion of tainted fish.

**Conclusion:**

Mast cell activation disease causes problems of increased complexity in children and adults. The increased prevalence and severity of mast cell activation disease has been attributed to dramatic changes in our lifestyles and modern living environments. These changes likely impact the integrity of the epithelial barriers, leading to loss of tolerance in the mast cell compartment. Here, we present a case of a nonatopic, 74-year-old female who developed mast cell activation disease after exposure to a potent environmental toxin. Mast cell activation disease commonly involves several organ systems, with patients often referred to a succession of different specialists. This results in delayed diagnosis and suboptimal care. Instead, early recognition of mast cell activation disease would lead to better outcomes. We review the literature, describing the diagnostic criteria for mast cell activation disorders that can improve recognition of this multiorgan system syndrome. Further research is needed into the interaction of epithelial barrier disruption and the dysregulation of the immune system.

## Case report

### Background

Mast cells are closely associated with epithelium, serving as sentinels responsible for the recognition of tissue injury and coordination of the initial inflammatory response. Upon detection of the injured cell content, mast cells then tailor the release of preformed and newly produced chemical mediators to the detected challenge, via an array of pathogen receptors. In addition to immunoglobulin E (IgE) receptor-triggered mast cell activation (MCA), commonly referred to allergic or atopic disorders, non-IgE mediated mast cell activation follows engagement of toll-like receptors (TLRs), immunoglobulin G (IgG) receptors, and complement receptors. Upon containment of the extrinsic challenge, acute inflammation is downregulated, and repair of the injured tissue ensues. The mast cell compartments must return to a baseline steady state, to remain tolerant towards self-antigens and harmless entities, including environmental conditions, to prevent unnecessary immune activation and chronic hypersensitivity disorders.

Over the past 50 years, an increasing number of patients are experiencing episodes of aberrant mast cell activation, not associated with allergen specific mast cell disease or systemic mastocytosis. This led to proposed diagnostic criteria of mast cell activation syndrome (MCAS). MCAS is a heterogeneous disorder, defined by a combination of (1) recurrent symptoms typical of MCA, (2) an increase of validated mast cell-derived mediators, and (3) response to treatment with mast cell stabilizing or MC mediator-targeted therapies. Onset of MCAS ostensibly reflects the loss of tolerance in the mast cell compartment to nonthreatening entities and nonhazardous environmental conditions. The etiology of chronic mast cell dysregulation and associated intolerance to self-antigens or harmless entities is not well understood, but a growing number of studies point to exposure of the epithelial borders, which leads to inappropriate or excessive mast cell activation or impaired resolution of acute inflammation following neutralization of the identified pathogen.

## Case presentation

A 74-year-old Caucasian woman, with a past medical history of arrhythmia, breast cancer with bilateral mastectomy, an acoustic neuroma, menopause, hypothyroidism, and chronic musculoskeletal complaints, presented to the emergency department (ED) with intense redness of her face and hands, burning sensation involving her face and neck, palpitations, a “pounding headache,” difficulty breathing, dysphagia, and a rapid heart rate. Her illness began shortly after eating a tuna burger from a local fish market. She denied previous incidents of food intolerance or anaphylaxis. In the past, the patient had only used prescription or over-the-counter antihistamines for drug induced urticaria, opiate pain relievers, and nonsteroidal antiinflammatory drugs (NSAIDs). Both pain relievers had been avoided for years.

Upon arrival in the ED, her vital signs were body temperature of 97.0 °F (36.1 °C), pulse of 120 beats per minute (bpm), blood pressure of 123/55 mmHg, oxygen concentration of 99% on room air, and a respiration rate of 16 breaths per minute. She also reported chest pain, with a level of 5/10. On physical examination, she was found to have an erythematous facial rash and complained of diffuse itching. The patient was administered prednisone 60 mg and diphenhydramine 50 mg, with relief of her headache, rash, chest pain, and pruritis. In addition, an electrocardiogram revealed sinus tachycardia, at 107 bpm, and she was ultimately diagnosed with a cardiac arrhythmia. No other laboratory tests were ordered. Two samples of the tuna steak were obtained from the local fish market, which sickened four adults with scombrotoxinism. The samples contained histamine levels of 500 ppm and 4850 ppm, both greater than the threshold of less than 50 ppm deemed nontoxic by federal regulation. Since both the patient and her husband exhibited allergic-like reactions to the tuna burger, scombroid poisoning was suspected.

Unlike her husband, who was treated and recovered from this episode of scombrotoxinism, the patient experienced persistent urticaria and angioedema, with the diagnosis of new onset dermatographism, following completion of the 5-day course of oral prednisone and diphenhydramine from the initial ED visit. Despite the use topical corticosteroids and daily levocetirizine 5 mg, loratadine 40 mg, and fexofenadine 180 mg, the patient experienced daily symptoms, for the next 6 weeks. The patient returned to the local emergency department for persistent facial swelling and difficulty swallowing following ingestion of crab cakes for dinner, which had been tolerated in the past. The patient reported difficulty swallowing, sensation of food getting stuck, increased throat clearing, and nasal congestion, as well as numbness and tingling of the tongue, lips, and throat. In the local ED, the patient was found to have a body temperature of 98.2 °F (36.8 °C), a pulse of 106 bpm, blood pressure of 140/65 mmHg, oxygen concentration of 98% on room air, and a respiration rate of 18 breaths per minute. On physical examination, an erythematous facial rash was reported, and the patient was evaluated by an otolaryngologist in the ED, given new onset dysphagia. The otolaryngologist reported erythema of the laryngeal mucosa. The patient was discharged with a course of famotidine 20 mg, prednisone 40 mg, and diphenhydramine 25 mg.

Following this visit to the ED, the patient underwent an evaluation by a local allergy specialist. Recurrent urticaria was noted, and laboratory tests revealed the following: total serum immunoglobulin E (IgE) of 16 IU/mL; total complement level within normal limits; undetectable serum IgE for insect venoms, tuna, and crab; undetectable autoantibodies to nuclear antigen (ANA); a negative recommended avoidance of all seafood.

The patient sought another opinion with our practice, regarding chronic urticaria and new onset shellfish allergy, 1 month after her last ED visit and 6 months after scombroid poisoning. She had continued her regimen of thrice daily histamine receptor-1 blockade and famotidine 20 mg twice daily. In addition to the recurrent flushing, rhinitis complaints, dyspepsia, and episodic dysphagia, the patient cited chest tightness at rest, in the evening, and on exertion. Spirometry revealed an obstructive pattern consistent with reversible airway disease, which exhibited a modest improvement following administration of levalbuterol 0.63 mg/3 mL. Another set of laboratory tests were ordered, with the following results: serum tryptase of 4.8 and a serum IgE 17 IU/mL, which were comparable to previous results, but a plasma histamine level of 13 nmol/L was detected (normal range 0–8 nmol/L) (Table 1).

**Table 1:** Results of serial testing of tryptase and histamine between 2014 and 2015

	Ref. range	12/20/2014	2/10/2015	2/12/2015	2/22/2015	8/7/2015
Tryptase	2–10 ng/mL	4.8	4.7	2.2	5.2	5.0
	Ref. Range	10/17/2014 15:09	12/17/2014 18:52	12/20/2014 03:59	2/11/2015 12:32	8/7/201 5 14:44
Histamine	0–8 nmol/L	13 (H)	< 8	16 (H)	< 8	< 8

The patient maintained a limited diet, given her history of new onset shellfish allergy, and carried an epinephrine autoinjector and a short-acting beta agonist inhaler for the anaphylaxis and asthma, respectively. The patient opted for treatment with omalizumab for chronic urticaria, and was prescribed 300 mg, administered every 4 weeks, since she was still symptomatic despite thrice daily antihistamines, twice daily leukotriene modifiers, and oral and inhaled cromolyn preparations.

Following her first dose of omalizumab, the patient complained of increased pruritis, flushing, and chest tightness, prompting another ED visit. The patient required epinephrine administration for a grade two, drug-induced anaphylactic event. Following another course of oral corticosteroids and diphenhydramine, the patient underwent a graded challenge to omalizumab. Over a 3-month period, omalizumab 150 mg, administered every 2 weeks, was successfully reintroduced [1]. As recently reported, the addition of omalizumab, is the standard care for patients with MCAS.

The diagnosis of MCAS in this patient, following scombroid poisoning, is supported by detection of increased mast cell derived mediators. Following anaphylaxis, to crab meat and then to the first administration of omalizumab, we tested for validated markers of mast cell activation within a few hours of each suspected MCA event (Table 1). An elevated serum histamine level was detected following ingestion of crab meat and her first omalizumab administration. After her third episode of anaphylaxis, serum histamine returned to baseline, but serum tryptase also reduced to 2.2 μg/mL from her previous recorded values, which ranged from 4.8 to 5.2 μg/mL. Along with the constellation of recurrent symptoms of mast cell activation, including chronic urticaria, asthma, and susceptibility to anaphylaxis, and a partial response to histamine and leukotriene blockade, the detection of an elevated plasma level following episodes of anaphylaxis, and a reduction of serum tryptase of greater than 2 μg/mL and > 20%, led to the diagnosis of nonclonal mast cell activation syndrome (MCAS) (Table 2).

**Table 2:** Proposed criteria for mast cell activation syndrome (all three criteria must be present) (Akin, 2017)

Mast cell activation disease (MCAD), includes individual mast cell activation disorders and mast cell activation syndrome (MCAS)	
Individual mast cell activation disorders	Skin: urticaria, angioedema, flushing
	Gastrointestinal: nausea, vomiting, diarrhea: abdominal cramping/pain, gastroesophageal reflux disease
	Cardiovascular: hypotensive syncope or near syncope, tachycardia
	Neuropsychiatric: brain fog, anxiety, depression, paresthesia, lightheadedness
	Respiratory: wheezing
	Naso-ocular: conjunctival injection, pruritus, nasal congestion, posterior rhinorrhea
	Genitourinary: pain with urination, urinary frequency; females—pain with intimate relations; heavy, painful menses
	Musculoskeletal: bone pain, muscle pain, degenerative disc disease, osteopenia/osteoporosis
MCAS diagnosis: three criteria	(1) In addition to two or more of the above-mentioned MCA disorders
	(2) An elevated biomarker for mast cell activation syndrome, which can include semi tryptase or urinary mediators, such as prostaglandin and histamine metabolites (prostaglandin 1)2, 11 Beta Prostaglandin F2 Alpha, or methylhistamine
	(3) A decrease in the frequency, severity, or resolution of symptoms with histamine receptor antagonists or other mast cell-targeted medications, such as ketotifen, omalizumab, cromones, or tricyclic agents

Upon reaching the maintenance dose of omalizumab 150 mg, every 2 weeks, the patient was able to expand her diet and reduce her daily medical regimen to control her asthmatic condition and chronic spontaneous urticaria. In addition to omalizumab, the patient is maintained on daily levocetirizine, chlorpheniramine, zafirlukast, and famotidine. Moreover, under ongoing treatment her quality of life was improved, including less disrupted sleep, resumption of outdoor exercise, reintroduction of food stuffs, except seafood, and less frequent urticarial outbreaks.

## Discussion

Over the past 50 years, there has been a rise in classic mast cell activation disorders, such as rhinitis, food hypersensitivity, anaphylaxis, urticaria, and asthma, as well as less well known MCAD, such as neuropsychiatric and musculoskeletal syndromes. A growing number of pediatric and adult patients are experiencing recurrent episodes of MCA, not associated with allergen-specific mast cell activation disease or systemic mastocytosis. This led to proposed diagnostic criteria of mast cell activation syndrome, which includes the following: (1) systemic mast cell activation involving two or more organ systems, in parallel; (2) an increase in validated mast cell markers during symptomatic episodes; and (3) having a major response to medications that block MCA or inhibit the action of MC-derived mediators (Table 2). The diagnosis of mast cell activation syndrome (MCAS) is appropriate, when all three criteria are met [2, 3].

Mechanisms contributing to rise of mast cell activation disorders, including MCAS, have not been established. Studies have implicated environmental exposures from modern living leading to excessive mast cell-initiated acute inflammation, or impaired resolution of inflammation following neutralization of the offending agent. This case report demonstrates the onset of MCAS in a 74-year-old woman following exposure to scombrotoxin.

Scombroid poisoning, or histamine fish poisoning, is a toxic event with symptoms and treatment that mimic anaphylaxis. Scombroid poisoning results from consumption of mishandled fish. Histamine and other decomposition products are generated in time-temperature abused raw fish by bacterial, enzymatic conversion of free histidine. The onset of scombroid poisoning is typically from 10 minutes to 1 hour following consumption of poisonous fish. The symptoms are variable and include peppery or metallic taste, oral numbness, headache, dizziness, palpitations, rapid and weak pulse (low blood pressure), difficulty in swallowing, and thirst. Scombroid poisoning often mimics allergen-specific IgE-triggered mast cell activation, with symptoms such as hives, rash, flushing, facial swelling, nausea, vomiting, abdominal cramps, and diarrhea. Less specific symptoms such as anxiety are less frequently observed [4].

Recovery is usually complete within 24 hours, but in rare cases can last for days. For example, serious cardiovascular compromise and respiratory complications have been observed, and in a few unusual cases, hospitalization, including treatment for anaphylaxis [5, 6]. There are two reports of patients developing chronic mast cell activation disorders following scombroid poisoning: after ingesting decomposed tuna, a 21-year-old woman, with a history of intermittent asthma, developed poorly controlled asthma, requiring multiple ED visits and five daily medications to control new onset, severe persistent asthma. Another woman, without a history of MCADs, was diagnosed with adult-onset rhinitis and asthma following scombroid poisoning [7, 8].

Scombroid poisoning is associated with elevated histamine levels in the outbreak-associated samples. However, there is not a clear dose-response relationship between oral administration of histamine and the magnitude of toxicity associated with scombroid poisoning [5]. Interestingly, several studies point to the impact of “histamine releasing factors” on the epithelium and closely associated mast cells, by enteric, gram-negative bacteria found in the fish cutis and intestines. These include *Escherichia coli*, *Klebsiella* species, *Pseudomonas aeruginosa*, and *Morganella morganii* [4]. As reported in animal models of anaphylaxis and asthma, loss of tolerance in the mast cell compartment can be induced by exposure to chemical adjuvants and microbial-derived substances, such as aluminum hydroxide and lipopolysaccharides, respectively [9–11]. Combined with signals of tissue injury, called alarmins or damage associated molecular patterns (DAMPs), the microbial-derived substances are potent stimulants of mast cell activation.

With this patient, the reportedly high levels of histamine detected in the two samples of tuna from this seafood market greatly exceeded the level of histamine deemed toxic by the US Food and Drug Administration (FDA), more than 50 mg/100 g of tuna (50 ppm). Such high levels of histamine ostensibly reflect the burden of scombrotoxins, which in turn induced epithelial damage and delivered large quantities of histamine to the poisoned diners of the tainted fish. That is, the combination of histamine and histamine release factors, scombrotoxins, may have either severely disrupted the epithelium border and consequently caused a pronounced MCA event, or interfered with the processes that resolve inflammation after the acute insult has been neutralized. Three of the four adults with scombroid poisoning made a full recovery within 24 hours with conservative medical management. In contrast, our patient, with a distant history of drug-induced urticaria, required daily medication for adult-onset spontaneous inducible urticaria/angioedema, hypersensitivity gastroenteritis, and moderate persistent asthma, starting in the seventh decade of life. In addition to diet restriction, including avoidance of seafood, gluten, cow’s milk, soybean, and nuts, she must carry an epinephrine autoinjector and short-acting beta agonist inhaler given her history of anaphylaxis and intrinsic asthma exacerbations, following scombroid poisoning [12].

## Conclusion

In summary, we describe the clinical presentation of a presentation of MCAS following scombroid poisoning. The diagnosis is appropriate given her combination of (1) typical symptoms, (2) laboratory abnormalities, and (3) response to treatment. Following scombroid poisoning, adult-onset chronic urticaria/angioedema evolved into susceptibility of anaphylaxis, chronic hypersensitivity gastroenteritis, and moderate persistent asthma. Symptomatic episodes were associated with an elevation in validated mast cell markers, both serum tryptase and histamine, and her symptoms came under better control with the addition of omalizumab, the anti-immunoglobulin E monoclonal antibody, to her daily regimen of cromolyn antihistamines and leukotriene blockade. Despite the rise of immediate hypersensitivity disorders, it is not uncommon for MCAS to be diagnosed late, as observed with our patient. This leads to delayed recommendations for more effective treatment prescriptions of medications, with adverse effects; both of which may contribute to progressive disease. As recently described, the greatest hurdle to diagnosis and management of MCAS is to correctly attribute appropriate symptoms to this disorder, and timely measurement of validated mast cell activation markers. The latter will differentiate MC activation disorders from other syndromes with overlapping symptomology [12, 13]. This case also provides insight into potential etiologies and risk factors for development of MCAS.

Mast cell dysregulation can be due to a mutation affecting homeostasis of the mast cell compartment, clonal MCAS (mastocytosis, hyper alpha tryptasemia), or extrinsic factors driving aberrant MCA. Although the etiology of this newly recognized hypersensitivity syndrome is unknown, the course of events leading to adult-onset MCAS in this patient points to the disruption of the epithelial barrier by scombrotoxins. This, in turn, disrupted the tight regulation of this tissue-based, innate immune cell population, and increased susceptibility to mast cell activation events. Animal models and epidemiological studies support this theory of recurrent or severe injury to the epithelium in susceptible children and adults leading to development of immediate and delayed hypersensitivity syndromes: disruption of the epithelial barrier leads to loss of regulation of the mast cell compartment. This, in turn, leads to increased susceptibility of unnecessary inflammation, from impaired barrier function, and implicates the use of therapeutics targeting epithelial–mast cell interactions in preservation or restoration of immune homeostasis [10, 14].

## Data Availability

The datasets used and analyzed during the current study are available from the corresponding author on reasonable request.

## Abbreviations

MC: Mast cells
MCAS: Mast cell activation syndrome
TLR: Toll-like receptors
IgR: Immunoglobulin receptors
CR: Complement receptors
ARDS: Acute respiratory distress syndrome
PAMPs: Pathogen associated molecular pattern
POTS: Postural orthostatic tachycardia syndrome
hEDS: Hypermobile type Ehlers–Danlos syndrome
PID: Primary immunodeficiency
ACE2: Angiotensin-converting enzyme 2
DAMPs: Damage-associated molecular patterns
